# Implementation of Girsanov Reweighting in OpenMM and Deeptime

**DOI:** 10.1021/acs.jpcb.4c01702

**Published:** 2024-06-12

**Authors:** Joana-Lysiane Schäfer, Bettina G. Keller

**Affiliations:** Department of Biology, Chemistry, and Pharmacy, Freie Universität Berlin, Berlin 14195, Germany

## Abstract

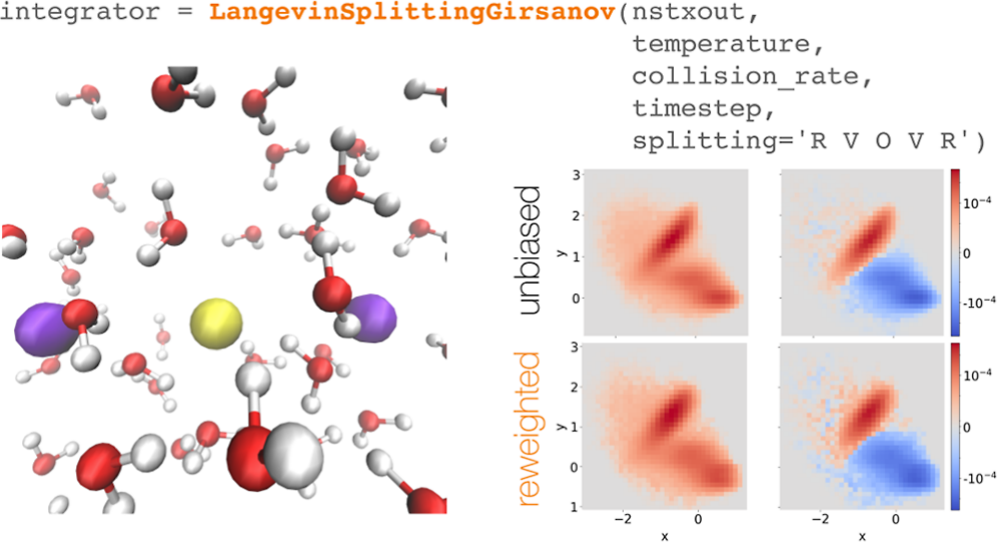

Classical molecular dynamics (MD) simulations provide invaluable insights into complex molecular systems but face limitations in capturing phenomena occurring on time scales beyond their reach. To bridge this gap, various enhanced sampling techniques have been developed, which are complemented by reweighting techniques to recover the unbiased dynamics. Girsanov reweighting is a reweighting technique that reweights simulation paths, generated by a stochastic MD integrator, without evoking an effective model of the dynamics. Instead, it calculates the relative path probability density at the time resolution of the MD integrator. Efficient implementation of Girsanov reweighting requires that the reweighting factors are calculated on-the-fly during the simulations and thus needs to be implemented within the MD integrator. Here, we present a comprehensive guide for implementing Girsanov reweighting into MD simulations. We demonstrate the implementation in the MD simulation package OpenMM by extending the library openmmtools. Additionally, we implemented a reweighted Markov state model estimator within the time series analysis package Deeptime.

## Introduction

Classical molecular dynamics (MD) simulations yield trajectories of a molecular system at atomistic resolution and are an excellent tool to study the dynamics of complex molecular systems. A complication emerges from the fact that relevant time scales of the molecular system, from slow conformational changes to complex formation and ultimately chemical reactions, occur on time scales that are orders of magnitude beyond the time scales that can be covered by an unbiased MD simulation. To close this time scale gap a wide variety of enhanced sampling techniques^1^ have been proposed, which broadly can be classified into methods which (1) change the temperature of the system,^2,3^ (2) change the classical Hamiltonian, in particular the potential energy function, of the system,^4−8^ or (3) bias the initial state of the trajectory but otherwise leave the dynamics unchanged.^9,10^ A necessary complement of enhanced sampling techniques are reweighting methods,^1,11,12^ which recover the unperturbed stationary density and the unperturbed dynamics from biased simulations.

Girsanov reweighting^13−20^ is a dynamical reweighting technique to recover the unbiased dynamics of a molecular system from simulations that were conducted with a biased potential. It differs from other dynamical reweighting techniques^21−25^ in that it does not assume an effective model of the molecular dynamics but reweights the dynamics directly on the level of the stochastic MD integrator. In this sense, Girsanov reweighting is an exact reweighting technique. The method is based on the theory of stochastic path integrals.^26−28^ It has been applied to model systems^13−17^ and peptide dynamics.^19^ Recently, it has been used for the machine learning of optimal collective variables based on enhanced sampling simulations.^29^

Girsanov reweighting can also be applied in a prospective manner. From MD trajectories obtained at a reference potential energy function, one can calculate the transition rates at a modified potential energy function and investigate the sensitivity of the transition rates with respect to certain parameters of the potential energy. In fact, while modern empirical potential energy functions often reproduce the structural and thermodynamic properties with high fidelity,^30,31^ the dynamic properties, such as transition times across barriers, may vary considerably across different potential energy models for the same molecule.^32^ In ref (33), Girsanov reweighting has been combined with the maximum caliber approach^34^ to optimize potential energy functions such that the resulting dynamics reproduce a specific transition rate.

Since Girsanov reweighting is based on stochastic path integrals, it requires that the MD simulations are carried out with a stochastic MD integrator.^35^ To achieve reweighting accuracy at the level of the MD integrator, the random numbers and bias force at each integration time step are collected and aggregated in path reweighting factors. The path reweighting factor can be calculated for any time interval [*t*, *t* + τ] of the entire MD trajectory and represents the relative probability of the path from *t* to *t* + τ at the target potential relative to its probability at the simulation potential.

Interpreting the path reweighting factor as the relative path probability gives an intuitive explanation of why stochastic MD integrators are needed for Girsanov reweighting. The probability of a specific deterministic path, by definition, is one at the simulation potential but zero at any other potential. Hence, the relative probability of a deterministic path, i.e., the ratio of the path probability between the target potential and the simulation potential, is always zero.

For stochastic integrators, the path reweighting factor can be calculated efficiently by formulating it in terms of the random numbers generated during a stochastic MD simulation and in terms of random number differences that account for the difference between the dynamics at the simulation potential and the dynamics at the target potential to which the simulated dynamics should be reweighted.^18,36^ The equation for the random number difference depends on the integrator and can easily be derived for the Euler-Maruyama integrator,^28,35^ of overdamped Langevin dynamics.^14−16^ However, a much more realistic description of the molecular dynamics is achieved by underdamped Langevin dynamics. Ref (37) (unpublished results) presents a general approach to derive the relative path probability density for stochastic integrators of underdamped Langevin dynamics, and it provides a framework to analyze whether the relative path probability for a given integration algorithm is defined throughout the path space. If the relative path probability density is defined, its value can be calculated, and reweighting underdamped Langevin dynamics is thus possible.

While specialized simulation protocols for Girsanov reweighting have been published, there is a lack of ready-to-use simulation programs that allow on-the-fly estimation of reweighting factors. Calculating the relative path probability density requires access to the random numbers and forces at the time resolution of the MD integrator. Even though the implementation of the relative path probability density amounts to adding a reporter to the inner loop of the MD integrator, for most simulation packages this requires deep knowledge of the package architecture. The goal of this contribution is to provide a template for efficiently implementing the relative path probability density into an MD program. We use OpenMM^38^ which provides an easy and versatile implementation of Langevin integrators via the openmmtools package,^39,40^ to demonstrate the implementation.

As an example analysis of the simulation data, we reweight Markov state models (MSMs)^41−46^ and implement the reweighting factor into the time-series analysis package Deeptime.^47^ While Girsanov reweighting can be used to reweight other rate estimators,^33^ MSMs have the advantage that they are well suited to model multistate dynamics and that, due to the short Markov lag time, a model of the long-time dynamics can be constructed from short paths. Our additions to the two software packages are open source and provide a blueprint for extending an MD simulation and analysis program to include Girsanov reweighting.

## Theory

### Molecular Simulations and Path Probabilities

Consider a system of *N* atoms with Cartesian position vector  and associated momentum vector . Momentum and position vectors can be combined to a phase space vector . The time-evolution of such a system in a thermal bath is modeled by underdamped Langevin dynamics
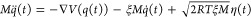
1The left-hand side of eq 1 represents the total force on the particles, where  is the acceleration vector at time *t*. *M* is the 3*N* × 3*N*-dimensional mass matrix, which contains the masses of the particles along its diagonal.

The force consists of three terms (right-hand side of eq 1): (i) the force due to the gradient of the potential energy function −∇*V*(*q*); (ii) the friction force, where  is the velocity vector and ξ is the collision rate with unit s^–1^; and (iii) a random force. According to the dissipation fluctuation theorem,^48^ the random force along the *l*th degree of freedom has the mean 0 and variance 2*RT*ξ*M*_*ll*_, where *R* is the ideal gas constant, *T* is the temperature, and *M*_*ll*_ is the *l* diagonal element in *M* and represents the mass associated with the *l*th degree of freedom. We model this random force by an uncorrelated white Gaussian noise with unit variance centered at 0, η(*t*), which is then scaled by  in order to fulfill the fluctuation dissipation theorem.^48^

We report the potential in molar energy units, J mol^–1^; correspondingly, the thermal energy is reported as molar quantity: *RT*. If energy units are used for the potential, *R* should be replaced by the Boltzmann constant *k*_B_ = *R*/*N*_A_ in eq 1 and all of the following equations. *N*_A_ is the Avogadro constant.

Langevin integrators^35^ are numerical schemes that provide an approximate solution to eq 1. They yield a time-discrete trajectory or path **x** = (*x*_0_, *x*_1_, *x*_2_, ...*x*_*n*_), where *x*_0_ = (*q*_0_, *p*_0_) is the initial state at time *t* = 0, *x*_*k*_ = (*q*_*k*_, *p*_*k*_) is the state at time *t* = *k*Δ*t* with time step Δ*t*. The path length is τ = *n*Δ*t*.

To model the random force, typical Langevin integrators draw either one or two random numbers from a standard normal distribution per integration time step and degree of freedom. The simulation thus additionally yields either one sequence of random number vectors, **η** = [η_0_, η_1_...η_*n*–1_], or two sequences of random number vectors, **η**^(1)^ = [η_0_^(1)^,η_1_^(1)^...η_*n*–1_^(1)^] and **η**^(2)^ = [η_0_^(2)^,η_1_^(2)^...η_*n*–1_^(2)^], where , , and . The *l*th element of a random number vector η_*k*_ contains the random number that determines the random force on the *l*th degree of freedom at the time step *k*.

The path probability  is the probability of observing a specific time-discretized path **x**. It can be decomposed using the Chapman–Kolmogorov equation^49^ as
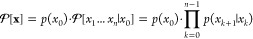
2where *p*(*x*_0_) is the probability density of the initial state. We here assume that the initial states are distributed according to the Boltzmann distribution
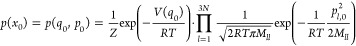
3where  is the classical configurational partition function,^50^ and *p*_*l*,0_ is the *l*th element of the initial momentum vector *p*_0_.

In eq 2, *p*(*x*_*k*+1_|*x*_*k*_) is the one-step transition probability to reach *x*_*k*+1_ within one integration step, given that the current state is *x*_*k*_. Langevin integrators that only take current state *x*_*k*_ into account when updating position and momentum implement a Markov process. For these integrators, eq 2 is exact. For Langevin integrators that additionally take the previous state *x*_*k*–1_ into account, eq 2 is an approximation.

The mathematical expression for *p*(*x*_*k*+1_|*x*_*k*_) depends on the Langevin integrator. One can however argue^36^ that the single-step transition probability is equal to the probability of drawing the random number vector η_*k*_ that gives rise to this specific transition
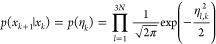
4where we used the random numbers η_*l*,*k*_ that are drawn from a standard normal distribution. Equation 4 holds for the Langevin integrator, which draws a single random number per integration step and degree of freedom *l*. The path probability density (eq 2) can then be written as
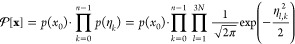
5Analogously, the path probability density (eq 2) for Langevin integrators that draw two random numbers per integration step and degree of freedom is
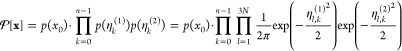
6

### Girsanov Reweighting

Girsanov reweighting is an importance sampling technique^1^ for path probability densities at different potential energy functions. Following the principles of importance sampling, a path expected value at target potential *Ṽ* can be reweighted as
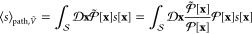
7where  is the path probability density at the target potential *Ṽ*(*q*),  is the path probability density at the simulation potential *V*(*q*). *s*[**x**] is a path observable, i.e., a function that assigns a real-valued number to each path **x**. ⟨...⟩ denotes an expected value, where we added the subscript “path” to emphasize that the expected values is calculated with respect to path probability density  and not with respect to a phase space probability *p*(*x*). The path integral  integrates over the space  of all possible time-discretized paths of length τ = *n*Δ*t*.

If an analytical expression for the relative path probability  can be found, the path expected value in eq 7 can be estimated from a set of paths S = (*x*_1_,...*x*_*n*_paths__) generated at the simulation potential *V*(*q*) as
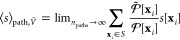
8

A critical question is under which conditions the relative path probability  exists. For time-continuous paths, these conditions have been discussed by Girsanov^27^ and Onsager and Machlup.^26^ It depends on the integrator in the case of time-discretized paths; ref (37) (unpublished reference) discusses path probability ratios for Langevin splitting operators.

The potential energy *V*(*q*), at which the simulation is carried out, and the target potential energy *Ṽ*(*q*), to which the path expected value is reweighted, are related by a potential *U*(*q*)
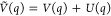
9Equation 9 takes the viewpoint of a perturbation,^18,20,33,36^ (ref (37) unpublished results), where the target potential differs from the reference (simulation) potential by a perturbation *U*(*q*). Alternatively, one can take the viewpoint of enhanced sampling simulations, where a bias potential *U*_bias_(*q*) needs to be subtracted from the simulation potential *V*(*q*) to obtain the molecular (target) potential, thus . By setting *U*(*q*) = −*U*_bias_(*q*), one can reconcile^19^ the enhanced sampling viewpoint with eq 9. It is important to be aware of the sign-convention since it enters the equations for the reweighting factor.

Using eq 5, the path probability density ratio can be expressed as^36^
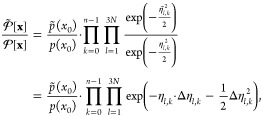
10where  is the random number vector that yields an update *x*_*k*_ → *x*_*k*+1_ at the target potential *Ṽ*(*q*), and η_*k*_ is the random number vector that yields the same update at the simulation potential *V*(*q*). The intuition is that one calculates  and compares it to the random number vector η_*k*_, which was used in the simulation at *V*(*q*). However, in our implementation,  is never calculated directly. Instead, the two random number vectors are related by a random number difference vector Δη_*k*_ with
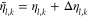
11The random numbers η_*l*,*k*_ can be recorded during the simulation at simulation potential *V*(*q*), but Δη_*k*,*l*_ needs to be calculated at each integration time step. The equation for Δη_*k*,*l*_ depends on the integrator, and the equations for various Langevin integrators are reported later in the text.

Analogously, using eq 6, the path probability density ratio can be expressed as^36^
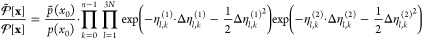
12with random number differences
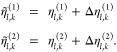
13The formulas for Δη_*l*,*k*_, Δη_*l*,*k*_^(1)^, and Δη_*l*,*k*_^(2)^ depend on the Langevin integrator^37^ (unpublished reference).

The path probability ratio can be decomposed into the probability ratio of the initial state  and the ratio of the path probabilities conditioned on the initial state
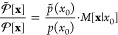
14where the conditional path probability ratio *M*[**x**|*x*_0_] is given by the product over time steps *k* in eqs 10 or 12. If the initial state *x*_0_ is drawn from the Boltzmann distribution eq 3, the probability ratio of the initial state in eqs 10 and 12 is given as
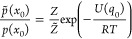
15

### Girsanov Reweighting for Langevin Integrators

Commonly used Langevin integrators are based on the splitting method.^51,52^Equation 1 is formulated as a vector field and separated in three terms, each of which is integrated separately, yielding three update operators,^35,51^ (ref (37) unpublished results) for the position *q*_*l*,*k*_ and the momentum 
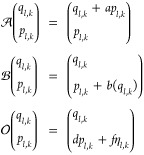
16where
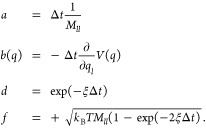
17We have formulated the three update operators here as operators that act on a single degree of freedom *l*. In the simulation of a multidimensional system, these update operators are applied to each degree of freedom.

A full update of *x*_*k*_ → *x*_*k*+1_ is obtained by sequentially applying all three update operators. Different Langevin integrators can be derived by varying the sequence in which the update operators are applied. Symmetric splitting integrators use a symmetric sequence. An example is the ABOBA algorithm, which applies the sequence , where update operators that appear twice in the sequence are applied with half a time step and are denoted with a prime. The corresponding parameters *a*′, *b*′(*q*), *d*′, and *f*′ are obtained by replacing Δ*t* with  in eq 18
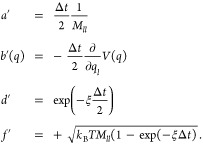
18

In eq 16, we formulated the updates for position and momenta, which leads to the ABO notation of the update operators.^51,53^ The update can analogously be formulated for position and velocities,^39,52^
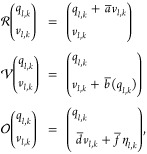
19where an -step is the analogue of an -step, a **V**-step is the analogue of a -step, and , , *d̅* = *d*, . Equation 19 leads to the RVO notation of Langevin splitting operators.^39,52^

A systematic approach to derive the random number difference between an update *x*_*k*_ → *x*_*k*+1_ at the simulation potential *V*(*q*) and the same update at the target potential *Ṽ*(*q*) is given in ref (37) (unpublished results). The reference also discusses why the relative path probability density  may not be defined for some Langevin splitting integrators. In these cases, path reweighting is not possible. For Langevin splitting integrators, which have a finite relative path probability density, the expressions for the random number differences are summarized in Table 1. The same equations for the random number difference are obtained when deriving them from the RVO update operators in eq 19. As an example, consider the RVO algorithm, whose random number difference is . But since *d̅* = *d* and , we obtain , which is the equation given in Table 1.

**Table 1:** Random Number Differences as Function of Perturbation Force  or Bias Force – ∇*U*_bias_, Integration Step Δ*t*, Dissipation *d*, and Fluctuation *f* Terms for Underdamped Langevin Integrators^a^

integrator	random number difference		integrator
(ABO)	Perturbation	bias	(RVO)
ABO	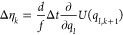	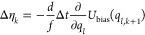	RVO
ABOBA	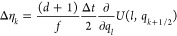	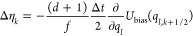	RVOVR
AOBOA	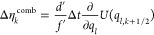	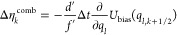	ROVOR
	η_*k*_^comb^ = *d*′η_*k*_^(1)^ + η_*k*_^(2)^	η_*k*_^comb^ = *d*′η_*k*_^(1)^ + η_*k*_^(2)^	
BOAOB	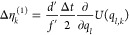	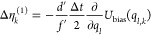	VOROV
	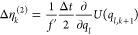	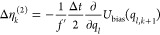	
OBABO/BP	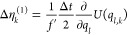	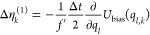	OVRVO
	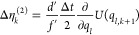	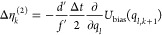	

a : perturbation force is evaluated before position update; : perturbation force is evaluated after position update; : perturbation force is evaluated at an intermediate position during update sequence of the Langevin integrator.

### Reweighting a Markov State Model

In a Markov state model (MSM),^41−46^ the 3*N*-dimensional position space Ω is discretized into *n*_state_ nonoverlapping states Ω_*i*_, i.e., . The probability vector **p**(*t*) contains the time-dependent probabilities *p*_*i*_(*t*) of finding the system in Ω_*i*_ at time *t*. The time-evolution of this discrete probability vector is then modeled as a Markov process
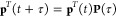
20where **P**(τ) is the MSM transition matrix with dimension *n*_states_ × *n*_states_. τ is the MSM lag time. Its elements  contain the conditional probability of finding the system in state Ω_*j*_ at time *t* + τ, given that it has been in state Ω_*i*_ at time *t*. By definition, the matrix is row-normalized, such that .

The conditional transition probability *P*_*ij*_(τ) can be calculated from the absolute transition probability *C*_*ij*_(τ) between states Ω_*i*_ and Ω_*j*_ as
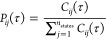
21

The absolute transition probability can be formulated as a path integral.^17,18^
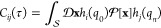
22where *h*_*i*_(*q*) is the indicator function of state Ω_*i*_
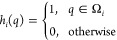
23Analogously, *h*_*j*_(*q*) is the indicator function of state Ω_*j*_. The path observable *s*[**x**] = *h*_*i*_(*q*_0_)*h*_*j*_(*q*_*n*_) thus evaluates to one if the path **x** starts in Ω_*i*_ and ends in Ω_*j*_ and to zero otherwise. *C*_*ij*_(τ) can be reweighted according to eq 8, and the reweighted estimator is
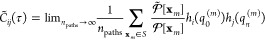
24where *S* = (*x*_1_,...*x*_*n*_paths__) is a set of paths of length τ = *n*Δ*t* generated at the simulation potential *V*(*q*), *q*_0_^(*m*)^ is the initial position of the *m*th path and *q*_*n*_^(*m*)^ is its last position.  is the absolute transition probability at a target potential *Ṽ*(*q*). Note that due to the normalization of the transition probability in eq 21, the constant ratio of the partition functions *Z*/*Z̃* appearing in eq 15 cancels.^18^

Once the reweighted MSM transition matrix **P̃**(τ) has been calculated, the MSM is analyzed via the eigenvalue and eigenvectors of **P̃**(τ)
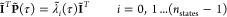
25where **l**_*i*_ is the *i*th left eigenvector and λ_*i*_(τ) is the associated eigenvalue. Due to the row-normalization of **P̃**(τ), the leading eigenvalue is always λ_0_ = 1 and its associated eigenvector **l**_0_ is the stationary density of the MSM. For a molecular system evolving according to eq 1, it should be equal to the (discretized) configurational Boltzmann density. The slow dynamic processes are represented by eigenvectors with eigenvalues close to 1.

In Markovian dynamics, the eigenvalues of the MSM transition matrix decay exponentially with the lag time τ: . Thus, if the time-evolution of **p**(*t*) can indeed be modeled by a Markov process, the implied time scale should be independent of τ
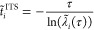
26The implied time scale test^43^ uses eq 26 to check the approximation quality of an MSM by calculating MSM transition matrices at various lag times and checking whether the right-hand side of eq 26 is indeed independent of τ.

## Methods

### General Approach

**Figure 1:**
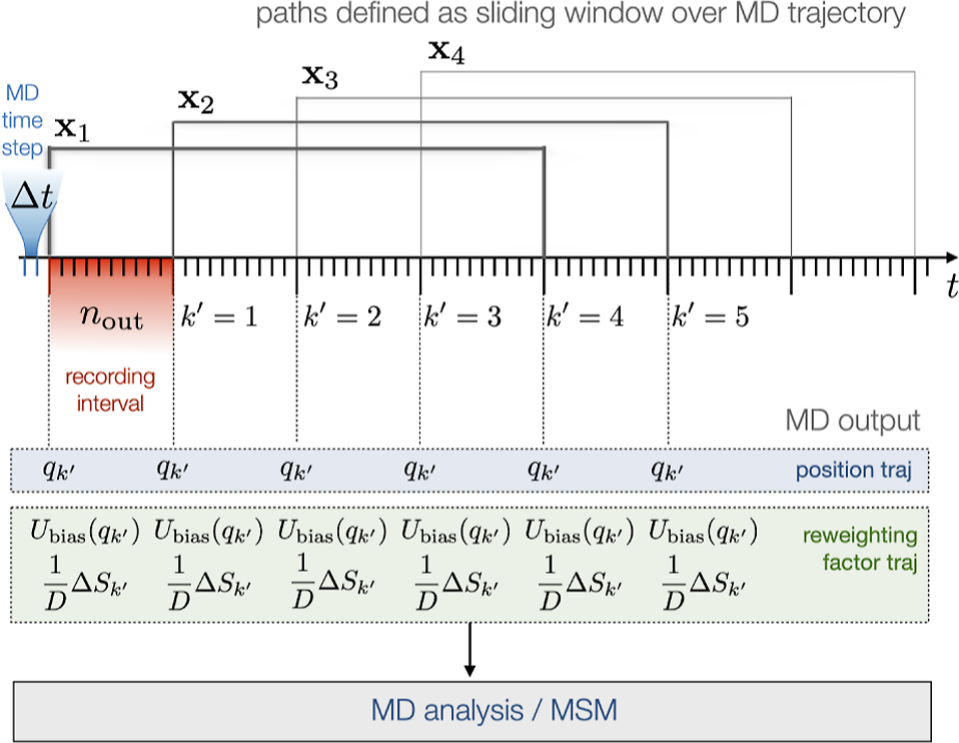
Overview of path reweighting.

Performing Girsanov path reweighting for MSMs requires two main steps: (i) computing the relative path probability (eqs 10 and 15) and (ii) estimating the reweighted MSM transition matrix (eq 24). The first step is handled by the MD simulation program on-the-fly during the simulation. The second step is handled by an MD analysis program. The data that is exchanged between the simulation and the analysis program are the position trajectory and an associated trajectory of path reweighting factors. Figure 1 illustrates the data flow.

In path reweighting, and in Girsanov reweighting in particular, the role of MD simulation is to generate a set of paths *S* = (*x*_1_,...*x*_*n*_paths__) at the simulation potential *V*(*q*). While it is possible to run a separate MD simulation for each path **x**_*i*_, in the context of MSMs the paths are usually extracted from a long trajectory via a sliding window. The path length is then τ = *n*Δ*t* = *n*_out_·*n*_interval_Δ*t*, where *n*_out_ represents the interval (in number of MD time steps Δ*t*) between writing coordinates to the output trajectory file, and *n*_interval_ is the number of intervals *n*_out_Δ*t* that fit into the path length τ. To illustrate, in Figure 1 coordinates are written to file every *n*_out_ = 10 time steps, and *n*_interval_ = 4 of these recording intervals fit into a path, yielding a path length of τ = 40Δ*t*. We denote the iteration of the MD integrator with index *k* and the index of the recorded coordinates by *k*′. The indices are related via *k* = *k*′*n*_out_.

path probability density is calculated and written to a file at the same interval as the coordinates. To do this efficiently, the relative path probability for a path starting at *k* = *k*′ = 0 is decomposed into a product of two factors as shown in eq 14. Inserting eq 15 and omitting the factor *Z*/*Z̃* yields
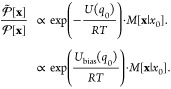
27We omitted the factor *Z*/*Z̃* because it cancels in the equation for the MSM transition probability (eq 21). The factor also cancels in other rate estimators.^33^

Using a sliding window, path **x**_*i*_ may start at any time-point *k* = *k*′*n*_*out*_ at which coordinates are written to file. The indices in eq 27 then shift accordingly, i.e., *q*_0_ → *q*_*k*′_ and [*x*_0_, *x*_1_...*x*_*n*_] → [*x*_*k*′_, *x*_*k*′+1_...*x*_*k*′+*n*_]. To be able to calculate the relative probability of the initial state of a sliding window path, the perturbation energy *U*(*q*_*k*′_) (or equivalently the bias energy *U*_bias_(*q*_*k*′_)) is written to the reweighting file, whenever coordinates  are written to file.

For a Langevin integrator which draws a single random number per degree of freedom and simulation time step, *M*[**x**|*x*_0_] is given by the double product in eq 10. To account for the recording interval, we reformulate the factor as
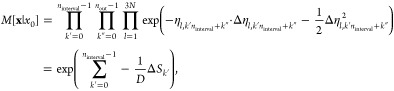
28where we split the product over the integration time steps *k* into an outer product over the recording intervals *k*′ and an inner product over the simulation time steps *k*″ within a given recording interval. The indices in eqs 10 and 29 are related by *k* = *k*′*n*_interval_ + *k*″. Furthermore, we have
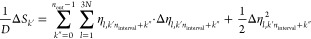
29 is calculated in the inner loop of the MD simulation program. At each integration time step *k* and for each degree of freedom, the random number  is recorded, and the random number difference  according to the appropriate equation in Table 1. The terms  are then summed over all degrees of freedom *l*, and the result is added to a buffer variable . The buffer variable is accumulated throughout the recording interval, i.e., for *n*_out_ steps. When the recording interval has elapsed and coordinates are written to the position trajectory, the value of the buffer variable  is written to the reweighting factor trajectory, and the buffer variable is reset to zero.

To calculate Δη_*k*_, one needs the bias force −∇*U*_bias_ or the perturbation force −∇*U*. When Girsanov reweighting is used to unbias a biased simulation, the bias force is already available within the inner loop of the simulation. When Girsanov reweighting is used in a prospective manner to predict the influence of a perturbation *U*(*q*) on a reference potential *V*(*q*), perturbation forces −∇*U*(*q*) need to be calculated in addition to the forces −∇*V*(*q*) that are used to propagate the system.

For Langevin integrators that draw two random numbers per integration time step,  is given as
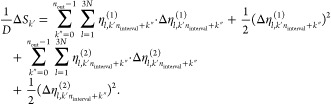
30

The variable name  is a nod to the path action, where *D* is the diffusion constant. When formulating the path probability in terms of the path action,^15,26,54^*S*[**x**], eqs 29 and 30 can be interpreted as the action difference between the simulation and the target potential, scaled by the diffusion constant.

Given the position trajectory at the simulation potential *V*(*q*) and the trajectory of path reweighting factors, an MSM at the target potential *Ṽ*(*q*) can be obtained via eq 24. The relative path probability density is calculated from the path reweighting factors by summing up  for all *n*_intervals_ recording intervals that make up the path **x** (eq 28) and multiplying the resulting conditional path reweighting factor *M*[**x**|*x*_0_] with the relative probability density of the initial state of the path (eq 27).

### Implementation in OpenMM via Openmmtools

**Figure 2:**
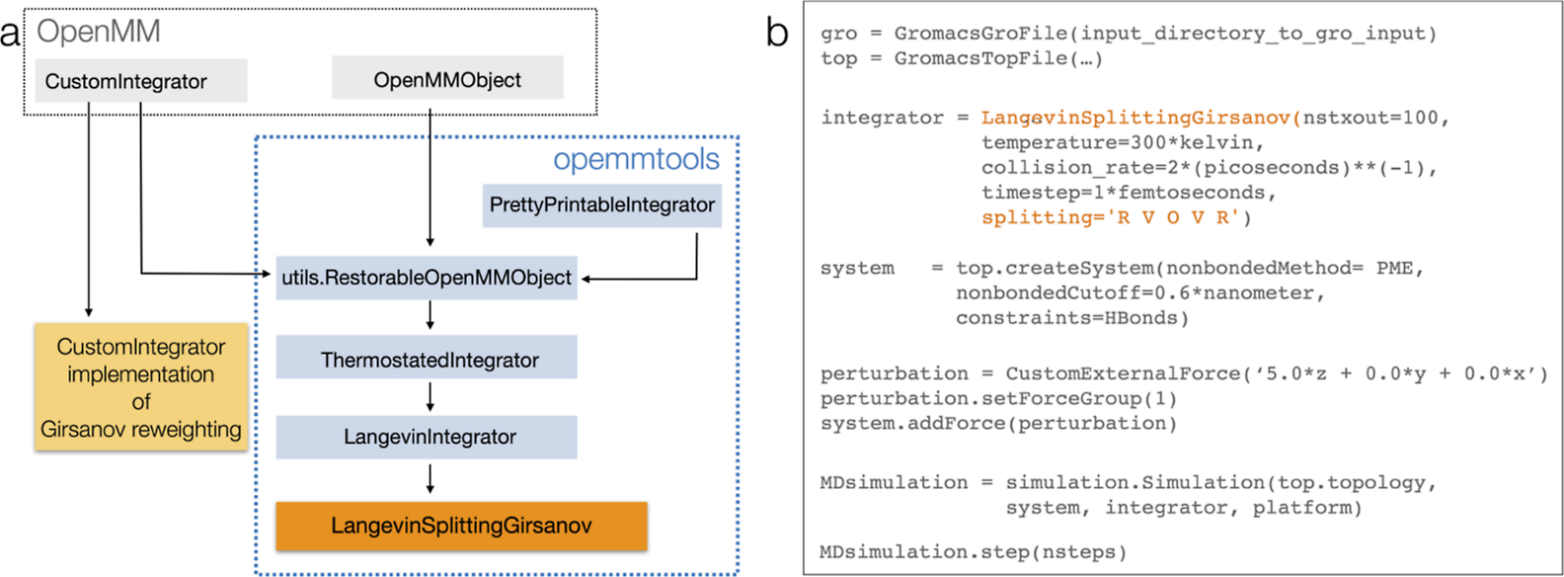
Implementation of the Girsanov reweighting in OpenMM via openmmtools. (a) Inheritance structure for the class LangevinSplittingGirsanov; (b) sketch of an OpenMM simulation script. LangevinSplittingGirsanov is called analogously to LangevinInegrator.

Openmmtools is a Python library layer that provides tools that allow for a user-friendly setup of complex simulation protocols. For our purpose, the most important openmmtools class is LangevinIntegrator. This class ultimately extends the OpenMM CustomIntegrator class (Figure 2) and provides access to Langevin Splitting integrators.^35,52,53^ Openmmtools used the RVO notation. The precise integrator can be conveniently specified by passing the sequence of update operators (eq 16) as a string. LangevinIntegrator implements the update operators as member functions _add_R_step(), _add_V_step(), and _add_O_step(). The sequence of update operations is realized by the function _add_integrator_steps(), which reads a string input, e.g., ‘R V O V R’ and outputs the corresponding update sequence.

To implement the reweighting factor trajectory, we created the new class LangevinSplittingGirsanov within openmmtools. With this new class, Girsanov reweighting simulations can be set up simply by specifying the Langevin integrator as a string, as one would normally do for a Langevin simulation with openmmtools. Figure 2b shows a sample OpenMM script for a Girsanov reweighting simulation.

LangevinSplittingGirsanov extends LangevinIntegrator and thus inherits all methods from LangevinIntegrator and its parent classes (Figure 2). In this way, variables for *M*_*ll*_, *T*, ξ, Δ*t*, etc. are automatically set. The random number differences Δη_*l*,*k*_ are implemented as a dispatch table, which can be accessed via the class function _delta_eta_table(self). When initializing the LangevinSplittingGirsanov with a string of update operators, the __init__-function checks whether Girsanov reweighting is possible for this particular Langevin integrator and whether the random number difference has been implemented. The variable  is provided by the function _get_logM(), which performs the summation over the 3*N* degrees of freedom, the recording interval *n*_out_, and, if necessary, the number of random numbers drawn per integration step. An additional factor sets the buffer variable for  to zero before the next recording step is performed.

An OpenMM simulation requires the input of an external force field file, which is processed to the OpenMM System object and can be called within the inner loop of the simulation; for more details cf. Figure S1. Additional bias forces can be provided by an interface like openmm-plumed^55^ and are stored in a separate force group of the OpenMM Force object. To ensure the bias force update at the integration step corresponding to the Langevin splitting scheme cf. Table 1, we have adapted _add_integrator_steps() to call the bias force group accordingly. Furthermore, we provide reporter class ReweightingReporter to record the path reweighting factors.

### Implementation in Deeptime

Deeptime^47^ is a Python library for the analysis of time series data and offers a wide range of tools to construct and analyze Markov models via the module markov. It implements estimation of a count matrix from a discretized trajectory via the function count_matrix_coo2_mult(···), which is provided by the module deeptime.markov.tools.estimation. The function currently implements the direct maximum-likelihood estimator for the matrix elements *C*_*ij*_(τ), which is obtained by setting the path probability ratio in eq 8 to one. We modified this function to implement the reweighted estimator in eq 24. The modified function accepts a trajectory of reweighting factors as an argument in addition to the discretized trajectory. If a trajectory of reweighting factors is passed to the function, the reweighted estimator is calculated; otherwise, it calculates the direct estimator. For each lag time τ, the reweighting factors are aggregated according to eqs 28 and 27 and used to calculate the reweighted count estimate according to eq 24.

The extended function is called by the class GirsanovReweightingEstimator, which is a child class of the class TransitionCountEstimator. The MaximumLikelihoodMSM class is available as a higher-level estimator of the MSM transition probabilities, which inputs the GirsanovReweightingEstimator, like the TransitionCountEstimator, and transforms the reweighted count matrix (21) into a transition matrix (eq 21). From there, all functionalities available in Deeptime, including the evaluation of the dominant eigenvalues (eq 25) and implied time scales (eq 26) for a series of lag times, can be achieved by feeding this transition matrix into the MarkovStateModel class.

## Results

In this section, the performance of the LangevinSplittingGirsanov class is investigated. Being part of the openmmtools package, the LangevinSplittingGirsanov class receives its forces from an OpenMM simulation object, which requires a molecular force field as the input (Figure 2). The LangevinSplittingGirsanov class thus cannot be directly compared to a reference implementation of a Langevin dynamics on an arbitrary low-dimensional potential. The OpenMM CustomIntegrator class can handle arbitrary low-dimensional potential as well as molecular force fields. To ensure consistency, we first compare the reference implementation to the OpenMM CustomIntegrator class using the Müller–Brown potential.^56^ Then we compare the OpenMM CustomIntegrator class to our LangevinSplittingGirsanov class by using a dissociation process in water.

### Müller–Brown Potential

**Figure 3:**
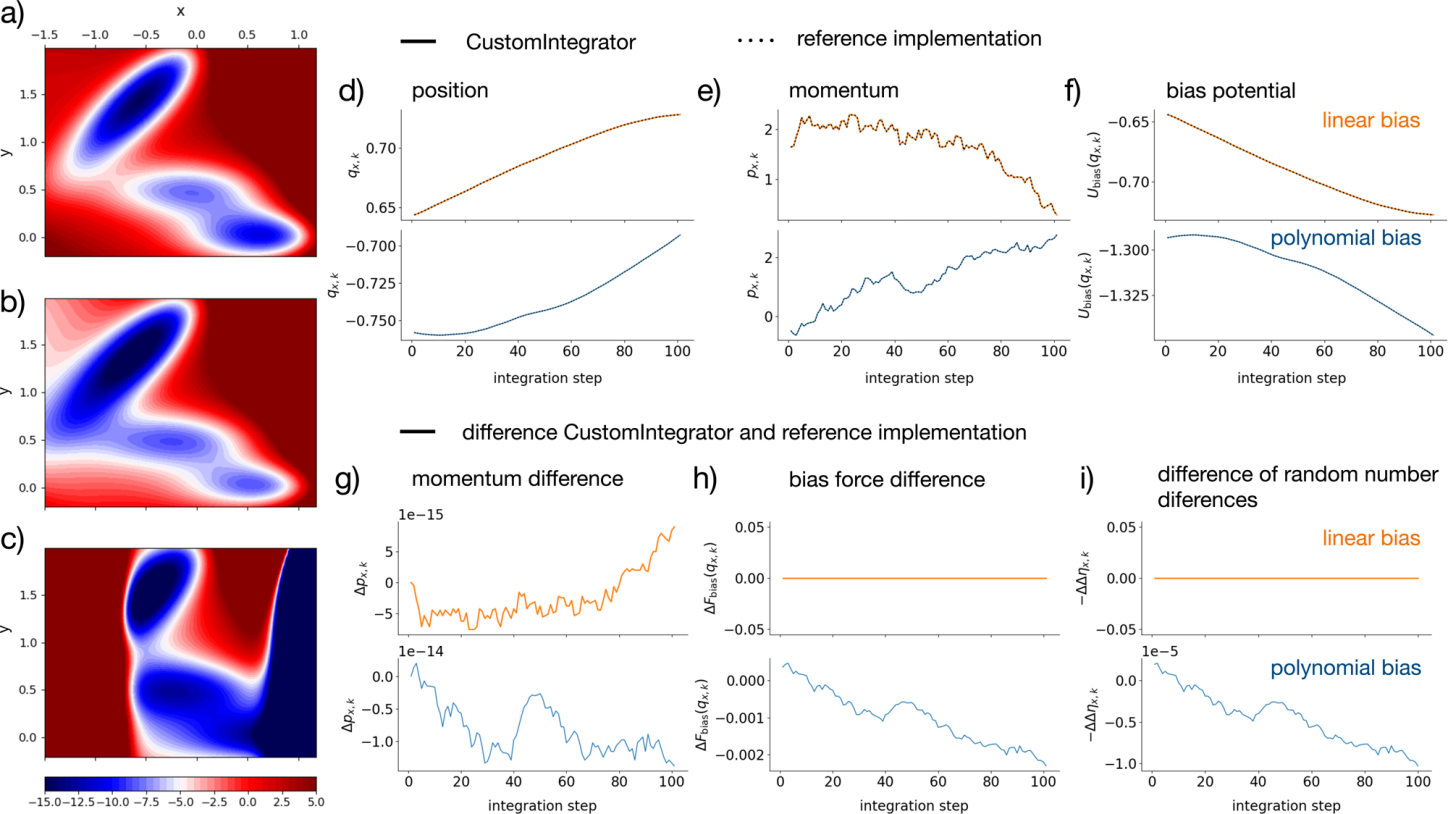
Comparison of the reference implementation (dotted) and the OpenMM CustomIntegrator class (solid) (a) Müller–Brown potential; (b) Müller–Brown potential and linear bias; (c) Müller–Brown potential and polynomial bias; Trajectories for 100 time steps of simulation: (d) *x*-component of the position, (e) *x*-component of the momentum, and (f) potential energy due to the bias. Difference between reference implementation and the OpenMM CustomIntegrator class (solid) for 100 time steps of simulation: (g) difference in *x*-component of ththe momentume momentum, (h) difference in the bias force , and (i) difference in the random number difference Δη_*k*_.

We use the Müller–Brown potential (56) (Figure 3a, parametrized according to eq 31 and Table 2) to compare our reference implementation to the implementation using the OpenMM CustomIntegrator class. The potential is characterized by a global minimum around (−0.5, 1.5) and two local minima around (0.0,0.5) and (0.5,0.0). The system was propagated on two biased potentials , where the first potential had a linear bias along *x* (Figure 3b), and the second potential had a strong polynomial bias along *x* (Figure 3c), cf. eq 32. The second form is motivated by a plumed-like bias, which is applied along a reaction coordinate and thus approximates a polynomial of the potential function. The linear bias was chosen to avoid position dependence in the force calculation. A consequence of applying the bias along *x* is that the path probabilities for the *y*-component of the path are the same in the simulation and the target potential.

**Table 2:** Parameters for Müller–Brown Potential

	*n* = 1	*n* = 2	*n* = 3	*n* = 4	units
*A*_*n*_	–20.0	–10.0	–17.0	1.5	kJ/mol
*a*_*n*_	–1.0	–1.0	–6.5	0.7	nm^–2^
*b*_*n*_	0.0	0.0	11.0	0.6	nm^–2^
*c*_*n*_	–10.0	–10.0	–6.5	0.7	nm^–2^
*x*_*n*_	1.0	0.0	–0.5	–1.0	nm
*y*_*n*_	0.0	0.5	1.5	1.0	nm

The reference implementation uses the analytical forces from this potential and the Langevin splitting integrator ABOBA^35^ to propagate the system. The OpenMM CustomIntegrator class uses numerical forces provided by OpenMM and also the Langevin splitting integrator ABOBA^35^ to propagate the system. Similarly, analytical bias forces are used to calculate the path reweighting factors  (eq 29) in the reference implementation, whereas numerical forces are used for this purpose in the OpenMM CustomIntegrator class.

Figure 3d–f shows the *x*-component of the position and momentum trajectory, as well as the trajectory of the bias energy for 100 simulation steps. We used the same random number sequence in both implementations. The simulations with the OpenMM CustomIntegrator class are shown as colored solid lines: orange for the linear bias and blue for the polynomial bias. The trajectories from the reference implementation are shown as black dotted lines. Visually the trajectories from the two implementations coincide. In fact, the difference between the two implementations is in the range of floating point precision. Figure 3g shows the difference of the momentum trajectories between the two implementations, which is on the order of 10^–14^ nm amu over 100 time steps, as an example. The difference of the position trajectory and the difference of the bias energy are shown in Figure S2. However, we do find that the bias force can differ in the two implementations (Figure 3h). While for the linear bias, analytical and numerical bias forces are identical (orange line in Figure 3h), the numerical force deviates from the analytical force for a nonlinear bias potential (blue line in Figure 3h). Since the bias force is used to calculate the random number difference, Δη_*k*_ differs between the two implementations for the nonlinear bias (Figure 3i).

In summary, apart from the difference between analytical and numerical forces, the OpenMM CustomIntegrator implementation reproduces the trajectories and path reweighting factor trajectories from our reference implementation.

**Figure 4:**
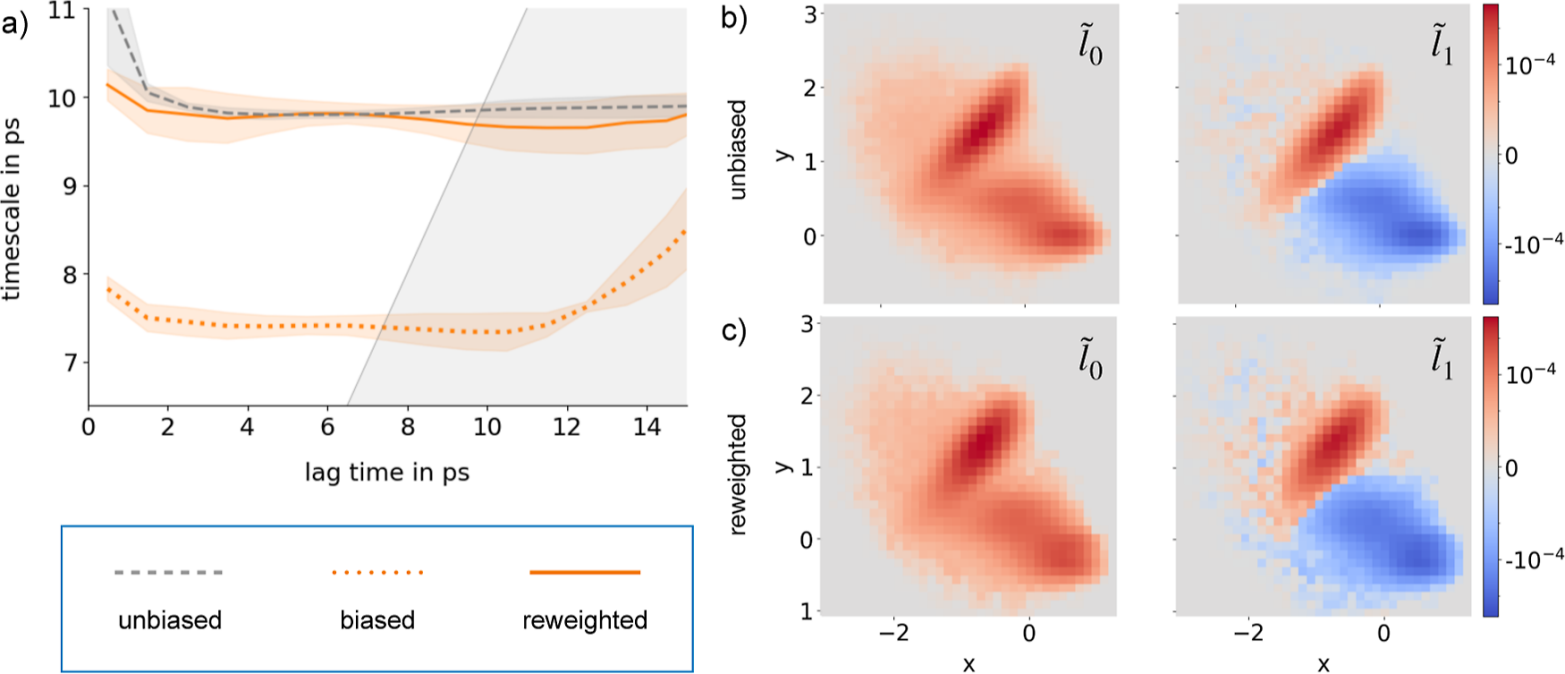
MSMs of the dynamics on the Müller–Brown potential. (a) Implied time scale  associated with the slowest process. (b) MSM eigenvectors  and  from an unbiased simulation on the Müller–Brown potential. (c) Reweighted MSM eigenvectors  and  from a simulation on the linearly biased Müller–Brown potential.

Next, we confirmed that we can reweight an MSM using the OpenMM CustomIntegrator class. Figure 4b shows the left dominant MSM eigenvectors  and  of the unbiased Müller–Brown potential, which is our target potential.  represents the stationary density of the Müller–Brown potential.  changes sign at the largest barrier of the potential and represents equilibration across this barrier. The MSM was obtained by a simulation at the unbiased Müller–Brown potential . Figure 4c shows the same eigenvectors obtained by simulating at a linearly biased potential  and then reweighting the MSM estimator (eq 24). The reweighted eigenvectors are in excellent agreement with the eigenvectors obtained from the unbiased simulation. Figure 4a shows the implied time scales of the MSM from the unbiased simulations (gray dashed line) and the MSM of the biased simulation without reweighting (dotted orange line) and with reweighting (solid orange line). Also for the implied time scales, the reweighted results agree very well with the results obtained from unbiased simulations.

### I^–^–Ca^2+^–I^–^ Dissociation Process in Water

**Figure 5:**
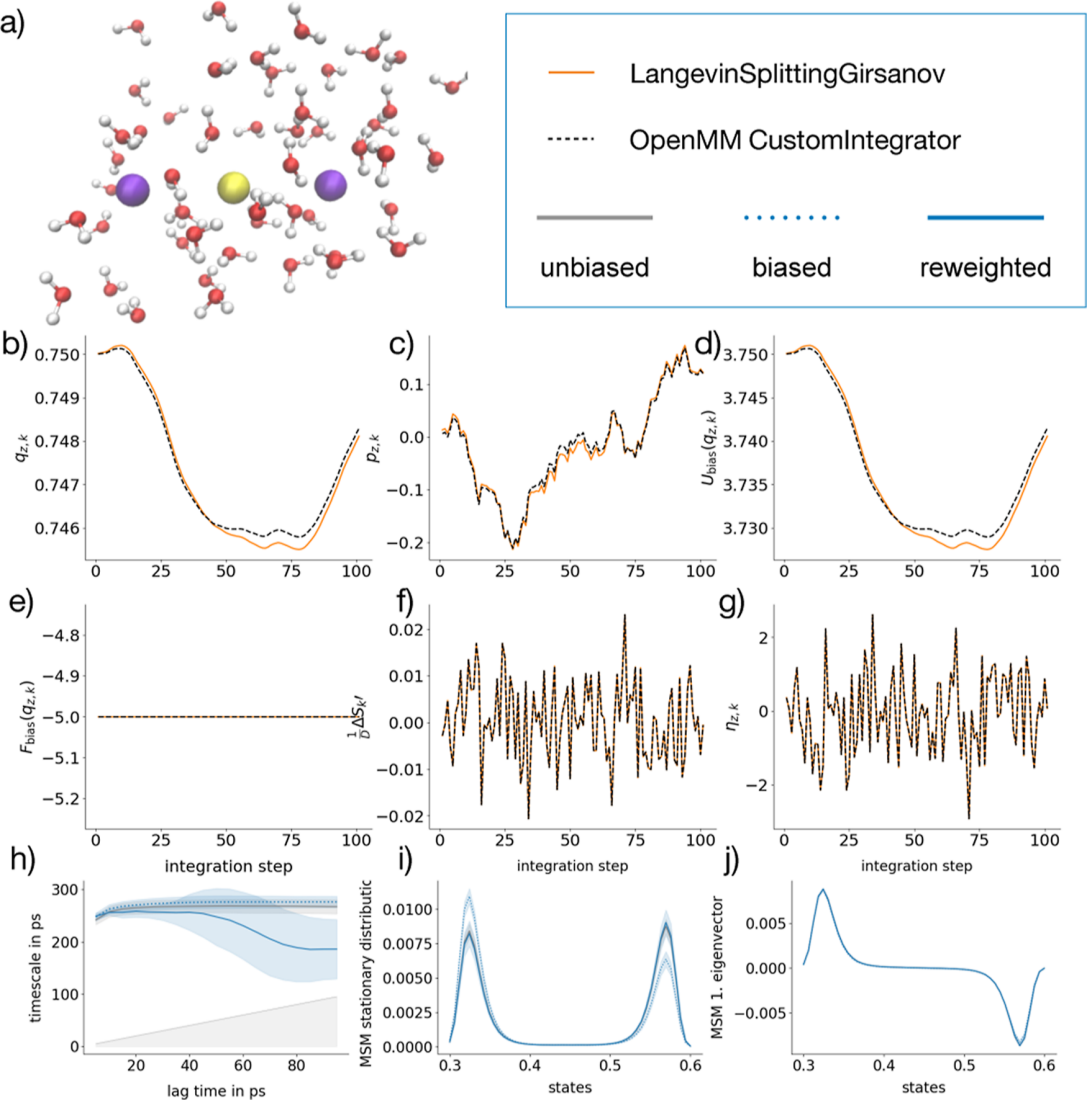
Molecular system of Ca^2+^I_2_^–^ ion pair in TIP3P water is shown in (a). Comparison between OpenMM CustomIntegrator (black dashed) and LangevinSplittingGirsanov class (orange solid) for 100 time steps of simulation: (b) position *q*_*z*,*k*_, (c) momentum *p*_*z*,*k*_, (d) bias potential *U*_bias_(*q*_*z*,*k*_), (e) bias force *F*_bias_(*q*_*z*,*k*_), (f) reweighting factors , and (g) random number η_*z*,*k*_. Implied time scales from I^–^–Ca^2+^–I^–^ reference simulation at a unbiased potential (gray solid), as well as from a reweighted MSM (solid) and a standard MSM (dotted) from linearly biased simulations with *k*_*z*_ = 5 kJ/mol/nm in blue (h). First (i) and second (j) left dominant MSM eigenvectors are shown, with the same color code used in (h).

We use simulations of CaI_2_ in TIP3P water (Figure 5a) to compare the implementation using the OpenMM CustomIntegrator class to our new LangevinSplittingGirsanov class (Figure 5b–g). The I^–^ anions were position restrained such that they maintained a distance of 0.9 nm along the *z*-coordinate. The Ca^2+^ cation was allowed to diffuse between these two anions, by applying medium strong harmonic restraints along the *y* and *x* coordinates of the Ca^2+^ cation. The resulting dynamics resembles a double-well dynamics along *z*, where the Ca^2+^ cation alternates between being bound to the I^–^ anion and being bound to the other I^–^ anion. In between the two bound states, there is a considerable free-energy barrier. The target potential *Ṽ*(*q*) consists of the molecular potential for this system including the above-mentioned restraints. In the potential , a linear bias potential along *z* is added, where *q* is the position vector of all atoms in the system.

Similar to the Müller–Brown potential (Figure 3), we compared the two implementations for a trajectory of 100 time steps, which was generated at the biased potential *V*(*q*). The two simulations use the same random number sequence for each degree of freedom. We observe a slight difference in the position and momentum trajectories between the OpenMM CustomIntegrator and the LangevinSplittingGirsanov. We suspect this can be attributed to slight algorithmic differences between our OpenMM CustomIntegrator implementation of the ABOBA algorithm and the implementation of the ABOBA algorithm via the parent class of LangevinSplittingGirsanov, the class LangevinIntegrator. Apart from this slight drift, the trajectory from our LangevinSplittingGirsanov class follows the trajectory from the OpenMM CustomIntegrator class very closely (Figure 5b–d).

Figure 5e–g shows trajectories of the bias force *F*_bias_(*q*_*z*,*k*_), of , and of the random number η_*z*,*k*_. The trajectories from the two implementations are identical for the linear bias (Figure S3a,b). Thus, the LangevinSplittingGirsanov implementation reproduces the trajectories and path reweighting factors from the OpenMM CustomIntegrator implementation.

Figure 5h–j tests whether we can reweight an MSM of the CaI_2_ dynamics using the LangevinSplittingGirsanov class. The MSM has been constructed by discretizing the *z*-coordinate, thereby neglecting the solvent degrees of freedom. Figure 5i shows the eigenvector  (gray solid line), which represents the stationary density at the target potential *Ṽ*(*q*), and the eigenvector *l*_1_ (blue dotted line), which represents the stationary density at the biased potential. Due to the bias potential, the two stationary densities differ. Reweighting the simulation data from the biased simulation recovers the stationary density  at the target potential *Ṽ*(*q*) with high accuracy. Figure 5j shows the eigenvectors  and *l*_1_, which represent the equilibration between the two bound states. Interestingly, the process seems to be insensitive to the bias, and thus the results from all three MSMs coincide. Figure 5h shows the associated implied time scales from simulations at the target potential *Ṽ*(*q*), simulations at the biased potential *V*(*q*), and simulations at the biased potential *V*(*q*) reweighted to the target potential *Ṽ*(*q*). While the MSM eigenvectors can be reweighted with high accuracy, the reweighted implied time scale exhibits a large statistical uncertainty. We will discuss possible reasons for this in Conclusions.

## Computational Details

### Müller–Brown Potential

The Müller–Brown potential^56^ is
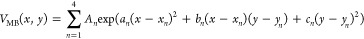
31its parameters are reported in Table 2.

We used two different bias potentials along the *x*-coordinate
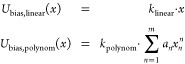
32with *k*_linear_ = 1 kJ/mol/nm and *k*_polynom_ = 50 kJ/mol/nm, *m* = 13, *a*_1_ = 2.06*e* – 02 nm^–1^, *a*_2_ = −2.32*e* – 02 nm^–2^, *a*_3_ = 3.83*e* – 03 nm^–3^, *a*_4_ = 3.92*e* – 02 nm^–3^, *a*_5_ = −1.39*e* – 02 nm^–3^, *a*_6_ = −3.39*e* – 02 nm^–3^, *a*_7_ = 3.82*e* – 04 nm^–3^, *a*_8_ = 1.24*e* – 02 nm^–3^, *a*_9_ = 3.37*e* – 03 nm^–3^, *a*_10_ = −1.18*e* – 03 nm^–3^, *a*_11_ = −7.50*e* – 04 nm^–3^, *a*_12_ = −1.38*e* – 04 nm^–3^, *a*_13_ = −8.81*e* – 06 nm^–3^. The particle mass was set to *m*_*p*_ = 1 amu. The ideal gas constant was set to *R* = 8.314 J/(K mol). The system was simulated using the Langevin splitting algorithm ABOBA^35^ with time step Δ*t* = 0.5 fs, friction coefficient ξ = 5 ps^–1^, and temperature *T* = 300 K.

We used two different implementations of ABOBA: (i) a reference stand-alone implementation in Python and (ii) an abstraction using the OpenMM CustomIntegrator class. Both implementations are available via GitHub.^57,58^

In the simulations using the reference implementation, the initial positions were drawn randomly from a uniform distribution and the initial velocities were set to *v*_0_ = (0.0, 0.0). We then generated 5 trajectories with 2.5 × 10^8^ time steps each for *V*_MB_(*x*, *y*) and 5 trajectories with 2.5 × 10^8^ time steps each for *V*_MB_(*x*, *y*) + *U*_bias,linear_(*x*).

In the simulations using the OpenMM CustomIntegrator class, the initial positions were drawn randomly from a uniform distribution, and the initial velocities were chosen according to the Boltzmann distribution at 300 K. We then generated 5 trajectories with 10^8^ time steps each for *V*_MB_(*x*, *y*), 5 trajectories with 10^8^ time steps each for *V*_MB_(*x*, *y*) + *U*_bias,linear_(*x*).

From the trajectories, we constructed two-dimensional MSMs on a grid with 36 states, where the *x*-dimension was discretized in the range −3.5 ≤ *x* ≤ 1.5, and the *y*-dimension was discretized in the range −1.5 ≤ *y* ≤ 3.5. The lag time was varied between 0.5 and 24.5 ps in steps of 1 ps. We calculated un-reweighted MSMs for the trajectories at *V*_MB_(*x*, *y*) and *V*_MB_(*x*, *y*) + *U*_bias,linear_(*x*) using the estimators provided by Deeptime.^47^ We calculated reweighted MSMs for the trajectories at *V*_MB_(*x*, *y*) + *U*_bias,linear_(*x*) using our implementation of the reweighted estimators which extends Deeptime.^57^

### I^–^–Ca^2+^–I^–^

The model system is a Ca^2+^I_2_^–^ ion pair (*m*_Ca_ = 40.08 amu, *m*_I_ = 126.90 amu) in an explicit TIP3P water box, containing 49 hydrogen bonds restrained water molecules. The nonbonded interaction parameters are chosen according to the AMBER99 force field,^59^ σ_Ca_ = 3.05 × 10^–1^, ε_Ca_ = 1.92, σ_I_ = 4.19 × 10^–1^, and ε_I_ = 1.67.

ABOBA simulations were performed either with openmmtools LangevinIntegrator^40^ or for biased runs with LangevinSplittingIntegrator.^58^ The initial positions were drawn randomly from a uniform distribution, and the initial velocities were chosen according to the Boltzmann distribution at 300 K. The step size of the integrator was 1 fs, with the friction coefficient of 2 ps^–1^. The particle mesh Ewald summation was used to calculate the interaction of all particle pairs within a cutoff of 0.49 nm.

Five trajectories with 2.5 × 10^8^ time steps each were created for the reference simulations at the unbiased potential. A total of 5 trajectories with 2 × 10^8^ time steps each were created at the biased potential.

To apply the bias linearly along one Cartesian coordinate of the Ca^2+^ ion
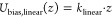
33with *k*_linear_ = 5 kJ/mol/nm, the degrees of freedom of the three atoms are restricted in the other two dimensions by a harmonic potential of the strength *k*_*x*_ = *k*_*y*_ = 200 × 10^3^ kJ/mol/nm. The displacement of the two iodide atoms is restricted in all directions, *k*_*z*_ = 500 × 10^3^ kJ/mol/nm. Restraining the degrees of freedom of the three atoms and the reduction to the collective variable of the iodide-calcium distance *d*_I–Ca_ means that the slowest process takes place on a double-well potential.

The trajectories were used to construct one-dimensional MSMs on a grid with 50 states of the iodide-calcium distance *d*_I–Ca_ in the range 0.3 ≤ *d*_I–Ca_ ≤ 0.6. The lag time was varied between 5 and 100 ps in steps of 5 ps. The unreweighted MSMs for both the reference and biased trajectories are calculated using the estimators provided by Deeptime,^47^ cf. Figure S4. The reweighted MSMs based on trajectories at a biased potential are performed with our implementation of the reweighted estimator.^57^

## Conclusions

This paper presents a guide for implementing Girsanov reweighting in MD simulation and analysis programs using the OpenMM with openmmtools and Deeptime as examples. In openmmtools, we extended an existing Langevin integrator class such that the reweighting factors are calculated on-the-fly and, at regular intervals, are written to a reweighting factor trajectory file. In Deeptime, we extended the MSM estimator class such that the transition counts are reweighted according to the reweighting factor trajectory. We demonstrated the correct functioning and error-free applicability of the newly implemented functions and classes using both a low-dimensional test system and a molecular system. The implementation can be readily used for larger systems. The extended software as well as instructions on how to use it are freely available.^57,58,60,61^

The computational cost of the Girsanov reweighting is usually small. During the simulation, it consists of recording the bias forces and energies and the random numbers for the degrees of freedom that are affected by the bias. During the analysis, it consists of evaluating eqs 29 or 30. Since the bias is typically low-dimensional, i.e., it affects only a few atoms in the simulation box,  is zero for most dimensions *l*, and the evaluation of the sums in eqs 29 and 30 incurs no relevant computational cost. In our example of calcium–iodine ion dissociation in water, the bias was applied along the *z*-dimension of the Ca^2+^ cation. Thus, the bias force was one-dimensional and the sum over the dimensions in eq 29 contained a single term. This is negligible compared to the cost of evaluating a single MD simulation step with a 450-dimensional force vector (49 water molecules and 3 ions in the simulation box).

A more critical question is under which circumstances Girsanov reweighting is efficient. One condition for efficiency is that the length of the reweighted paths has to be considerably shorter than the slow processes in the system. MSMs are one way to construct Girsanov-reweighted kinetic models from short paths because in these models the path length is equal to the MSM lag time.^18,19,36,62^ This is the approach we used here. Alternatively, one can reweight transition rates within the framework of transition path sampling or transition interface sampling, in which case only the typically short transition paths are reweighted.^33^ The second factor that influences the efficiency of Girsanov reweighting is the bias. Finding an optimal bias is closely connected to optimal control theory and to finding an optimal reaction coordinate along which the bias is applied.^29,63^

When reweighting MSMs, the reweighted dominant eigenvectors tend to be more accurate than the reweighted implied time scales.^18,36^ This might be due to several reasons. First, the relative path probability decreases exponentially with an increasing path length, falling below the numerical floating point accuracy. One simple remedy is to use a library which allows for a higher precision in the floating point numbers when analyzing the reweighting factor trajectories (for generating the path reweighting trajectory, the usual double precision should be sufficient). Second, in high-dimensional systems, the bias might shift the transition path ensemble for transitions across the barriers. A shifted transition path ensemble at the biased potential is then not representative of paths at the unbiased potential, which causes the relative path probability to be analytically close to zero. To avoid a shift in the transition paths, the bias can be deposited exclusively in the minima of the potential energy function.^22^ Finally, drifting implied time scales with large statistical uncertainties also occur in discrete Markov models which are estimated from unbiased simulations. This effect might be magnified by reweighting. More accurate Markov models are obtained by using an arbitrary ansatz function instead of a crisp discretization, such as tICA-Markov models,^64,65^ variational Markov models,^66^ or core-set Markov models.^45,62,67^ Since it is straightforward to reweight the estimators for these Markov models by the Girsanov relative path probability density, using suitable ansatz functions instead of crisp states will likely improve the accuracy of the reweighted implied time scales.

OpenMM provides very clean and clear access to the inner loop of the MD simulation via the CustomIntegrator class, and in previous studies, we have taken advantage of this class to implement Girsanov reweighing.^18,19,36^ However, this approach requires a detailed understanding of both the numerical schemes used to implement Langevin splitting integrators and the equations for the relative path probability. With the LangevinSplittingIntegrator class presented here, the running of a Girsanov reweighting simulation is simplified to one line of code in the OpenMM simulation script. A simple and error-resistant way to set up a Girsanov reweighting simulation is the starting point for more reweighting studies on large molecular systems.

## Data Availability

We provide the software for MD simulation with Girsanov reweighting as a pull request to the original openmmtools and openmm repository. added class LangevinSplittingGirsanov: https://github.com/choderalab/openmmtools/pull/729, added class ReweightingReporter: https://github.com/openmm/openmm/pull/4533. The extensions of the deeptime^47^ package for Girsanov reweighted Markov state models are also available as a pull request. modified class count_matrix_coo2_mult and added GirsanovReweightingEstimator: https://github.com/deeptime-ml/deeptime/pull/290. All simulation scripts and input files can be viewed in our reweightingtools repository. https://github.com/bkellerlab/reweightingtools.

## Supporting Information Available

Illustration of simulation software setup with tools for Girsanov path reweighting; representation of difference in x-dimension of 100-steps long trajectories; difference between the OpenMM CustomIntegrator class and LangevinSplittingGirsanov for 100 time steps of simulation; and dynamical properties of the I^–^-Ca^2+^-I^–^ system (PDF)
