# Supplementary material for: Implementation of Girsanov Reweighting in OpenMM and Deeptime
Source: J Phys Chem B. 2024 Jun 12;128(25):6014–27. doi: 10.1021/acs.jpcb.4c01702 (PMC11215775; doi:10.1021/acs.jpcb.4c01702)
Supplement: Supplementary file 1 — jp4c01702_si_001.pdf [file jp4c01702_si_001.pdf]

Implementation of Girsanov reweighting in OpenMM and Deeptime

Joana-Lysiane Schäfer and Bettina G. Keller*

Department of Biology, Chemistry, and Pharmacy, Freie Universität Berlin, Berlin 14195 Germany

E-mail: bettina.keller@fu-berlin.de

Supporting Information

Implementation

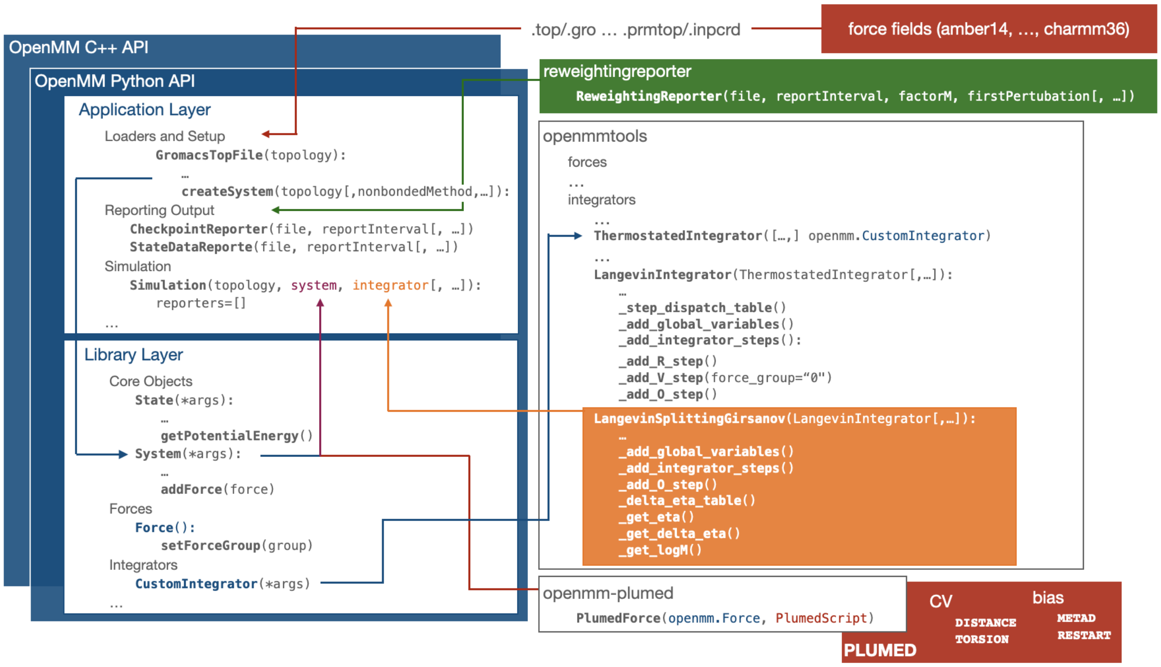

Figure S1: Illustration of simulation software setup with tools for Girsanov path reweighting. All relevant methods are shown in bold and their interaction can be followed using the colored arrows. Blue units represent the OpenMM package, red units mark inputs defining the force field. Important openmmtools or openmm-plumed functionalities are summarized in grey boxes. Orange unit highlights the additions to the openmmtools LangevinIntegrator and green the additional reweighting reporter.

Müller-Brown potential

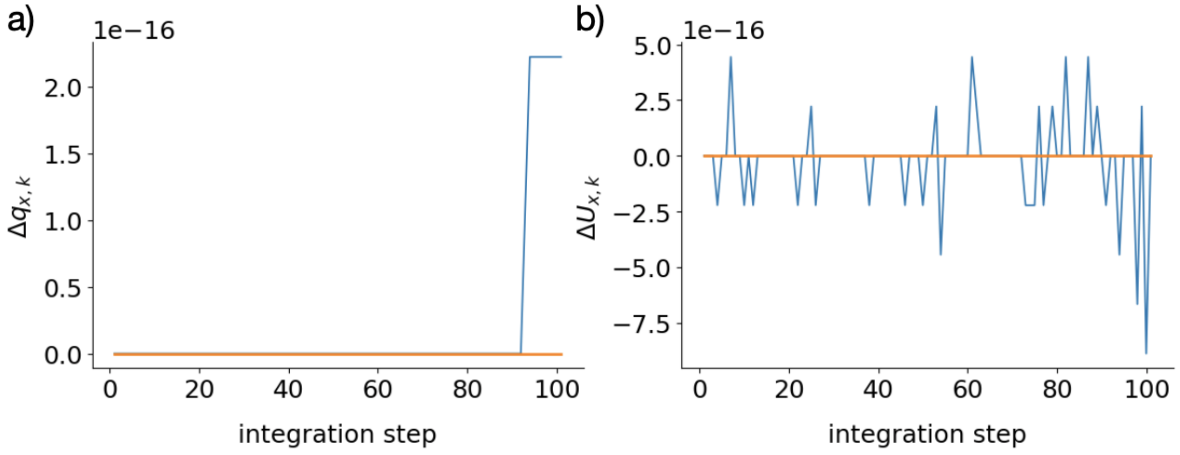

Figure S2: Representation of difference in x-dimension of 100-steps long trajectories of position q_x a) and bias potential b) for polynomial bias in blue and for linear bias in orange.

I^- - Ca^{2+} - I^-

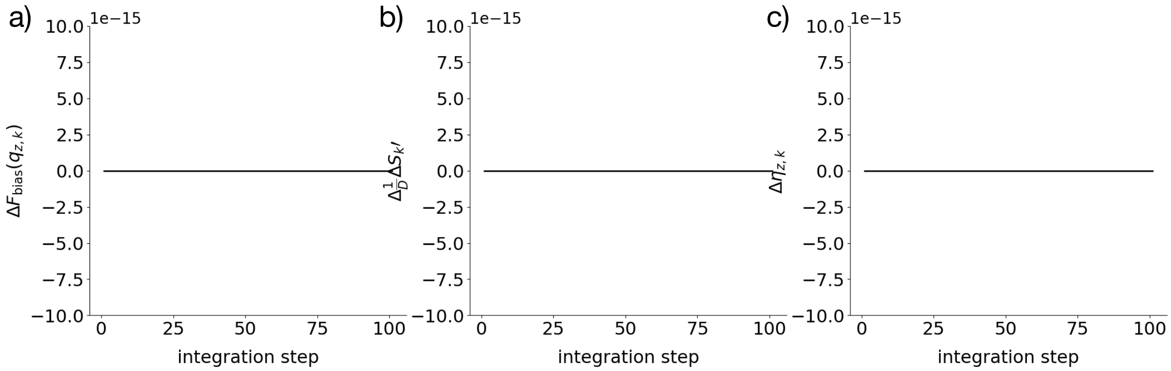

Figure S3: ICaI. Difference between the OpenMM CustomIntegrator class and LangevinSplittingGirsanov for 100 time steps of simulation: bias force $F_{\text{bias}}(q_{z,k})$ a), reweighting factors $\frac{1}{D}\Delta S_{k'}$ b) and the random number differences $\Delta\eta_{z,k}$ c).

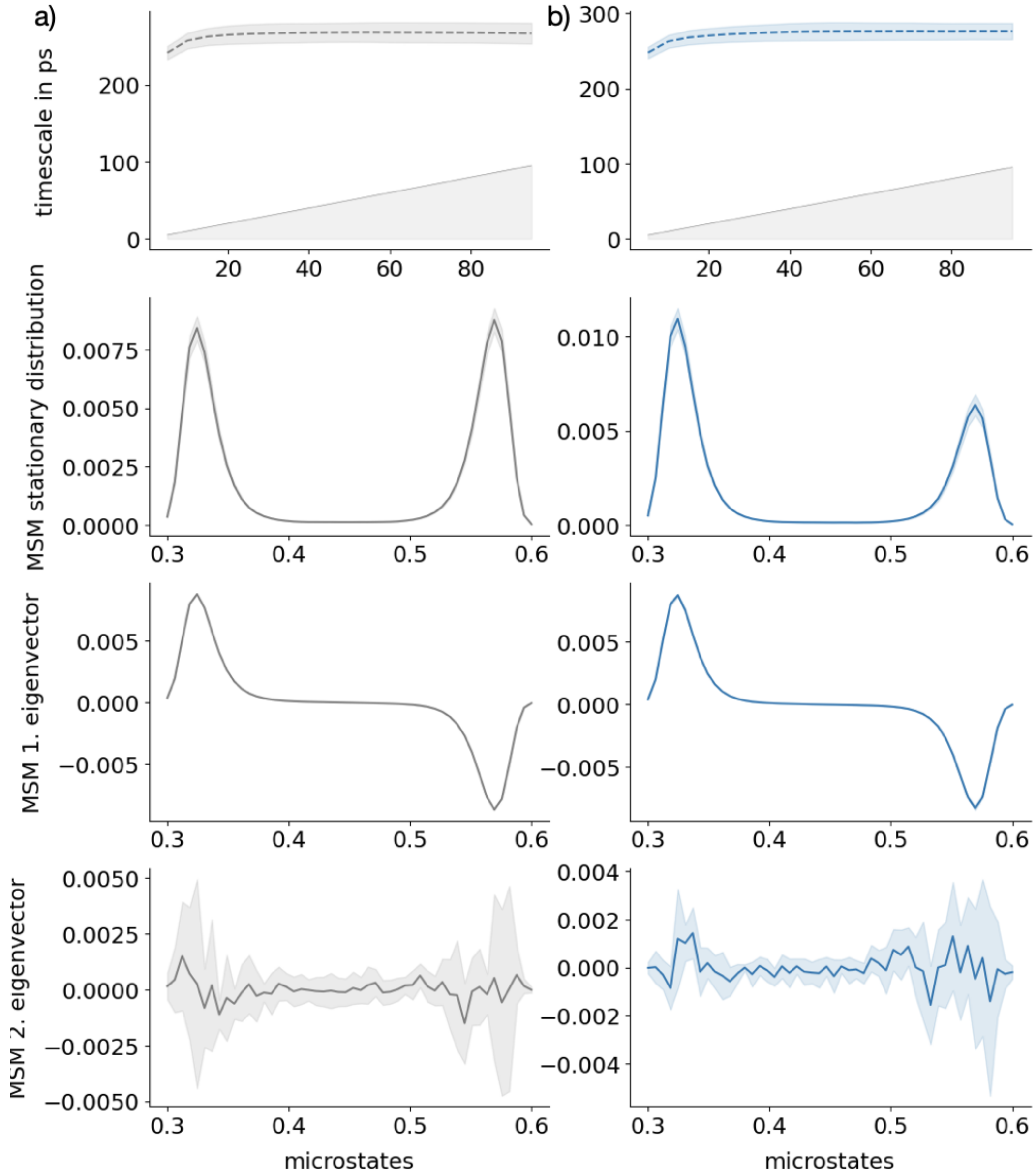

Figure S4: Dynamical properties of the I^- - Ca^{2+} - I^- system are shown from a reference simulation at a unbiased potential (grey) in a), as well as from a standard MSM from simulations at a linear biased potential with $k_z = 5$ kJ/mol/nm (blue) in b).
